# Evaluation of Dissipation Behavior, Residues, and Dietary Risk Assessment of Fludioxonil in Cherry via QuEChERS Using HPLC-MS/MS Technique

**DOI:** 10.3390/molecules26113344

**Published:** 2021-06-02

**Authors:** Shunyu Yao, Zixi Zhao, Wang Lu, Xin Dong, Jiye Hu, Xiaolu Liu

**Affiliations:** School of Chemistry and Biological Engineering, University of Science and Technology Beijing, Beijing 100083, China; yaoshunyu3d21@163.com (S.Y.); zhaozixi0612@163.com (Z.Z.); 18810861030@163.com (W.L.); dxzyfx@163.com (X.D.); jyhu@ustb.edu.cn (J.H.)

**Keywords:** fludioxonil, cherry, HPLC-MS/MS, dissipation behaviour, residues, dietary risk assessment

## Abstract

The chemical fungicide fludioxonil is widely used to control post-harvest fungal disease in cherries. This study was implemented to investigate the dissipation behaviours and residues of fludioxonil on cherries. A reliable and efficient analytical method was established. Cherry samples from four product areas were analyzed by QuEChERS and HPLC-MS/MS methods with acceptable linearity (R^2^ > 0.99), accuracy (recoveries of 81–94%), and precision (relative standard deviation of 2.5–11.9%). The limits of quantification (LOQs) and limits of detection (LODs) of cherries were 0.01 mg/kg and 0.005 mg/kg. The dissipation of fludioxonil on cherries followed first order kinetics with half-lives of 33.7–44.7 days. The terminal residues of fludioxonil were all lower than 5.00 mg/kg, which is the MRL recommended by the European Commission. According to Chinese dietary patterns and terminal residue distributions, the risk quotient (RQs) of fludioxonil was 0.61%, revealing that the evaluated cherries exhibited an acceptably low dietary risk to consumers.

## 1. Introduction

**Figure 1:**
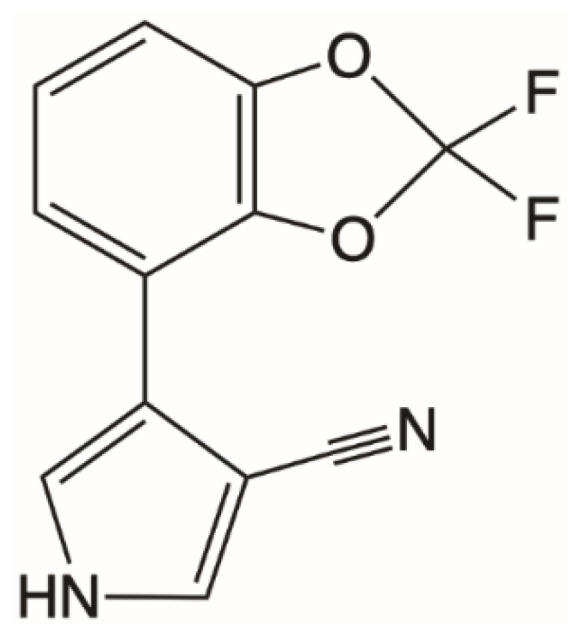
Chemical structures of fludioxonil.

Cherry (*Cerasus pseudocerasus Lindl.*) is one of the most popular and appreciated temperate fruits, not only for its sensory and nutritional properties but also for its content in bioactive compounds [1]. Over the last 16 years, global sweet cherry production has increased from 1.9 to 2.32 million tons [2]. Cherries are packed with antioxidants, vitamins, carbohydrates, and minerals that have beneficial effects on health [3]. However, postharvest diseases, mainly gray mold caused by *Botrytis cinerea*, have resulted in serious global economic losses in cherry and other berry fruits [4]. As a phenylpyrrole fungicide, fludioxonil is widely applied to control gray mold on cherries and other plants worldwide [5]. Fludioxonil is a 3-cyano-4-phenylpyrrolanalog of pyrrolnitrin with broad-spectrum activity against fungal plant pathogens among ascomycetes and basidiomycetes (Figure 1) [6]. It is highly effective when applied singly or in combination with other fungicides and is excellent in preventing mold decay by inhibiting both mycelial growth and spore germination [7]. As a non-systemic, surface fungicide, fludioxonil is registered for treatment at the pre- and post-harvest stages on leaves, fruits and seeds.

With the increasing global useof fludioxonil in the management of fruits and vegetables [8], it is commonly reported to have biocidal properties in that the effect of it involves a wide array of aquatic organisms which include the sensitivity of invertebrates and algae, the composition of microbial communities, and the feeding rate of Gammarus fossarum [9]. Fludioxonil also disrupts the expression of mi-RNA and cell motility, inhibits estradiol-induced cell proliferation in human breast cancer cells, and results in losses in membrane potential and ATP production in glial and neuronal cells [10,11]. The toxicity could be greater, as mixture with other pesticides showed observable toxicity to aquatic life, including zebrafish (Danio rerio), and caused the marked alteration of the activities of total superoxide dismutase (T-SOD) and catalase (CAT) [12].

Therefore, the toxicity of fludioxonil at relevant concentrations and dietary intake risk have attracted people’s attention to its safety. There are several studies on the residues of fludioxonil in grapes [13], tomatoes [14], and chrysanthemum [15] in field; and citrus [16], nectarines, apricots, and peaches [17] when it was the preservative. However, there are rare reports about the residues and dissipation behaviour of fludioxonil on the matrix of the cherry as an indoor preservative. It is urgent to evaluate the dietary intake risk of fludioxonil to different populations and monitor its residual dissipation and distribution. At present, the methods of detecting fludioxonil residues include liquid chromatography (LC) [18], gas chromatography (GC) [19], and cELISA [20]. Compared with other methods, the LC method analyzes samples faster and has a wider range of detectable compounds.

The aims of this work were (1): to establish a highly sensitive and selective detection method that can measure fludioxonil in cherries; (2): to investigate the dissipation behaviours and terminal residues of fludioxonil; and (3): to evaluate the potential risk of fludioxonil exposure through the dietary intake of cherries based on the residual level. The results provide a reference for the application of prevention and control of cherry gray mold. They also provide data for the modification of the maximum residue limit (MRL) values and the recommended dosage of these pesticides.

## 2. Results and Discussion

### 2.1. Optimisation of the LC-MS/MS Conditions

The fludioxonil standard solution was injected directly to compare the instrument response in positive and negative electrospray mode. Choosing the correct ionization mode can select the best (i.e., the most sensitive) ionization conditions for a given set of analytes. In many cases, ESI- is the better option owing to its improved sensitivity (ionization efficiency) and its potential for lower detection limits [21]. In this study, the negative mode was found to offer higher precursor signal intensities and better fragmentation patterns than the positive mode; consequently, negative ESI mode was selected for fludioxonil analysis. MS/MS scanning was then carried out, and two characteristic ions with prominent and stable responses were selected as daughter ions. The cone voltage and collision energy of the characteristic ions were optimized again. The resulting mass spectrometry parameters are shown in Table 1.

**Figure 2:**
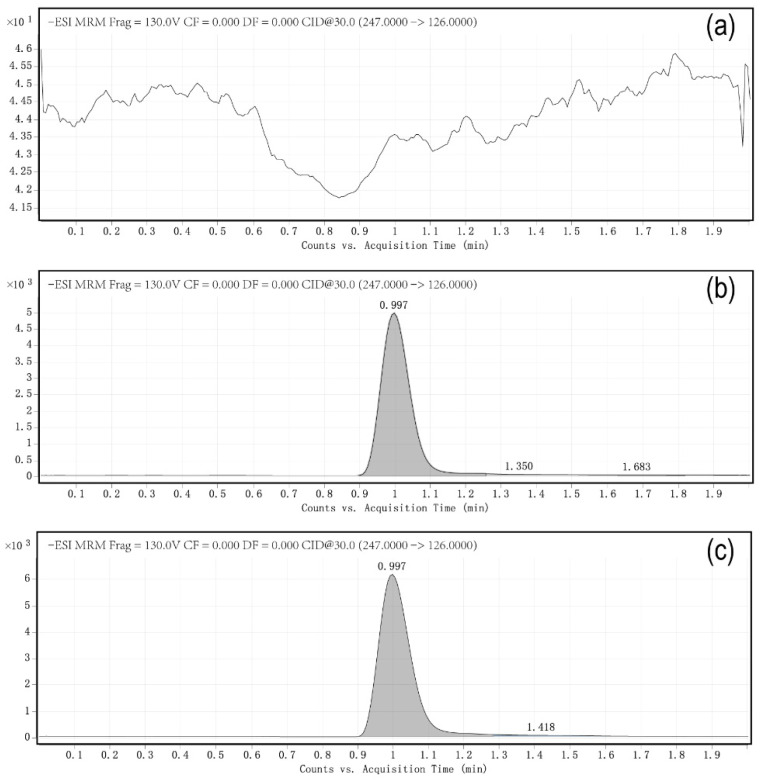
The liquid chromatography tandem mass spectrometry (LC-MS/MS) chromatogram of fludioxonil (**a**) sample of untreated cherries (0 mg/kg), (**b**) sample of fludioxonil standard in the cherry matrix (1.0 mg/kg), and (**c**) sample of cherries soaked in fludioxonil (4.93 mg/kg).

Optimal mobile phase combination could highly improve peak shapes and retention behaviours of the compounds in the LC system. Methanol and acetonitrile were selected as the most common dissolution media based on their effective solubility [22]. In previous studies, the use of pure methanol as the dissolution agent caused the column to produce two separate peaks, and the target compound exhibited poor retention behaviour. Similarly, pure acetonitrile as the dissolution agent resulted in bad peak shape and low sensitivity. In this study, it has been proven that the effect was the best when 0.2% acetic acid and 5 mM/L ammonium acetate were added to ultra-pure water. Figure 2 shows representative chromatograms of fludioxonil in (a) samples of untreated cherries, (b) samples of fludioxonil standard in the cherry matrix, and (c) samples of cherries soaked in fludioxonil. Among them, sample (a) was cherries soaked in clear water after harvest for control treatment, and Sample (b) added fludioxonil standard to the blank cherry sample artificially. The purpose was to eliminate the interference of the matrix effect on detection and to carry out an additive recovery experiment. Sample (c) was cherries soaked with the fungicide fludioxonil after harvest. As Figure 2 shows, the retention time of fludioxonil was 1 min. There were no apparent endogenous interference peaks. The target analytes matched the retention time of the standard sample exactly, and it did not co-elute with any other peaks.

In previous studies, the retention time of fludioxonil in a single sample was in the range of 5–12.75 min [13,15,18,23], while the retention time of fludioxonil in this method was only 1 min. It can be seen that when a large number of samples need to be processed, this method can improve work efficiency by shortening the average detection time of a single sample. In this sense, the method has the advantages of being fast and efficient.

### 2.2. Optimization of Extraction and Purification

Sample pre-treatment can go a long way to reduce the matrix effect during the analysis of pesticides in food matrices. Several sample pre-concentration procedures have been proposed for pesticide residue determination in fruits and vegetables, including: solid phase extraction (SPE), solid phase micro-extraction (SPME), micro-solid phase extraction (µ-SPE), and microwave assisted extraction (MAE) [24]. While these methods are highly efficient, they generally require considerable investment in instrumentation and allow a limited scope of pesticides that can be extracted under certain conditions. In 2003, Anastassiades et al. developed the QuEChERS method to process samples in pesticide residue analysis [25]. QuEChERS was widely used in the pesticide residue extraction process of samples with a high water content, such as fruits and vegetables [26]. Compared with the traditional standard extraction methods, the QuEChERS method has the following advantages: (1) high recovery rate; (2) high precision and accuracy that can be calibrated by internal standard method; (3) a wide range of pesticides that can be analyzed; (4) a fast sample processing speed; (5) the low amount of solvent, the low pollution, and the low price; and (6) the easy experiment operation and simple equipment [24,27,28]. Therefore, we chose the QuEChERS method for sample processing. The extraction efficiency of different solvents and adsorbents were two critical processes for the analysis of pesticide residues in different matrixes [29]. In general, acetonitrile was used as an extraction solvent because of the better extraction efficiency and less matrix interference [30]. Previous studies have shown that acetonitrile is an effective solvent for the extraction of fludioxonil. The recovery rates of standard addition in grapes and soil are 85.81–102.94% and 92–106.86%, respectively, and the relative standard deviation is less than 7% [23].

In the QuEChERS method, various purification procedures following extraction were necessary to remove co-extractives and interferences, which can improve the signal-to-noise ratio (S/N) of the target analytes [31]. PSA, GCB, C18, and MWCNTs were the most extensively used solid sorbents in dispersive solid-phase extraction (d-SPE) procedures [32,33]. During the purification process, the ideal sorbent formulation will only have a low impact on the target analytes [34]. PSA is mainly used to remove certain polar impurities, such as organic acids, fatty acids, and sugars [31]. C18 is mainly used to remove non-polar substances such as lipids, whereas GCB can effectively remove pigments in the matrix [35,36]. Considering that the cherry samples contained less fat content and that the main impurities were pigments and carbohydrates, GCB was used for purifying the target compounds. Considering that GCB can absorb specific pesticides [37], we chose to reduce its use content to ensure the recovery rate of the experiment while satisfying the purification effect. The results showed that 10 mg GCB and 150 mg anhydrous MgSO_4_ achieved a satisfactory clean up effect in the cherries.

As the final step of purification, the material and pore size were considered when choosing the filter. A nylon filter has high mechanical strength, can withstand most organic solvents, including acetonitrile, and has good chemical stability. The pore size of 0.22 μm is small enough to filter out most impurities other than the target compound and meets the experimental requirements [38]. Therefore, we chose the nylon 0.22 μm filter to extract pesticides from the cherry samples in our research. This method had the advantages of simple and quick experimental operation, low professional difficulty, low drug consumption, low pollution, low cost, and was not time consuming.

### 2.3. Method Validation

The study developed a simple HPLC-MS/MS method for quantifying fludioxonil residue in cherries. Analytical method validation was carried out according to the *Guidelines on Pesticide Residue Trials* [39], which included the following parameters: linearity, limits of quantitation (LOQs), limits of detection (LODs), accuracy, and precision.

Linearity was evaluated using R^2^, a good fit for the linear regression model, which was derived from a five-point standard curve; the standard curves were obtained by plotting the peak area against the corresponding concentration of target analytes [34]. The standard regression equation was y = 22,531x + 5196.1. Linearity over the concentration range of 0.005–5 mg/kg had a coefficient of 0.9955.

The limit of quantitation (LOQ) was defined as the lowest concentration satisfying the validation criteria for accuracy and precision. The limit of quantitation (LOQ) was 0.01 mg/kg, which was determined by multiple additive recovery tests. It was confirmed that the limit of quantitation (LOQ) was significantly lower than the maximum residue level (MRL) developed by the Ministry of Agriculture of China [40]. Thelimit of detection (LOD) was 0.005 mg/kg.

The average recovery and RSDs of five replicates of the cherry matrix was determined at spiked levels of 0.01, 0.1, and 5 mg/kg to verify and evaluate the accuracy of the method. The recovery (extraction efficiency) was calculated by dividing the peak area of an analyte from a pre-extraction spiked sample by the peak area of an analyte from a post-extraction spiked sample [41]. The results showed that the average recoveries of fludioxonil in cherries were 81–94% (Table 2). The relative standard deviations ranged from 2.5 to 11.9% as shown in Table 2. According to the provisions of the *Guidelines on Pesticide Residue Trials* (NY/T 788-2018) published by the Ministry of Agriculture, P. R. China, when the concentration range of the added drug is 0.01 mg/kg–0.1 mg/kg, the recovery rate should be 70–120%, RSD ≤ 20% [39]. The recovery test results showed that the analytical method had good linearity and reliability and could accurately detect fludioxonil.

### 2.4. Method Comparison

Various analytical methods, including LLE-GC-MSD, LLE-HPLC-DAD, QuEChERS-LC-MS/MS, Dilution-cELISA, QuEChERS-GC-NPD, QuEChERS-GC-MS, and QuEChERS-UPLC-MS/MS have been used to determine the fludioxonil residues in white grape juice, wine, apple juice, grapes, strawberries, and chrysanthemum. This work developed a fast and efficient method for detecting fludioxonil residues in cherries and compared several parameters of previous methods to analyze their differences in accuracy and precision (Table 3). Mercader et al. reported an cELISA method for the qualitative analysis of fludioxonil residues in apple juice where the LOD and the LOQ were 0.00006 mg/L and 0.005 mg/L, which is much more sensitive [20]. However, this method requires overnight incubation. Compared with those previously reported methods of GC, the QuEChERS-LC-MS/MS method has a lower LOD and LOQ as well as higher recovery and sensitivity, and the entire experimental process is much shorter [15].

Similar methods were also widely used in the detection of other pesticide residues in cherries. A previous study found that the QuEChERS method coupled with LC/MS/MS can effectively detected the residues of acetamprid in cherries, and the average recovery was from 80.12 to 98.04% [43]. A combination of solid phase microextraction (SPME) and LC/MS was used to determine the residues of five fungicides (trichlorfon, fluchondrion, o-phenol, pretilachlor, and toluene) in cherries. The samples were effectively separated, and the average recovery rate was satisfactory [44]. The eight highly polar pesticide residues (aminomethylphosphonic acid, N-acetyl-AMPA, chlormequat chloride, ethephon, glyphosate, ammonium glufosinate, N-acetyl-glufosinate, and maleic hydrazide) in cherries could be determined by liquid chromatography-triple quadrupole mass spectrometry (LC-MS/MS). Average recoveries ranged from 70.2 to 105.1%, and LOQ values ranged from 1.77 to 12.13 μg/kg [45]. Therefore, the LC/MS method is suitable for detecting multiple pesticide residues in cherries. This study optimized the sample preparation method and HPLC–MS/MS method and developed an advanced method to extract and detect fludioxonil residues in cherries. The RSDs and extraction recovery of the proposed method are satisfactory. It has a wide linear range, short sample preparation time, and detection time, which greatly improves the work efficiency.

### 2.5. Dissipation of Fludioxonil in Cherries

**Figure 3:**
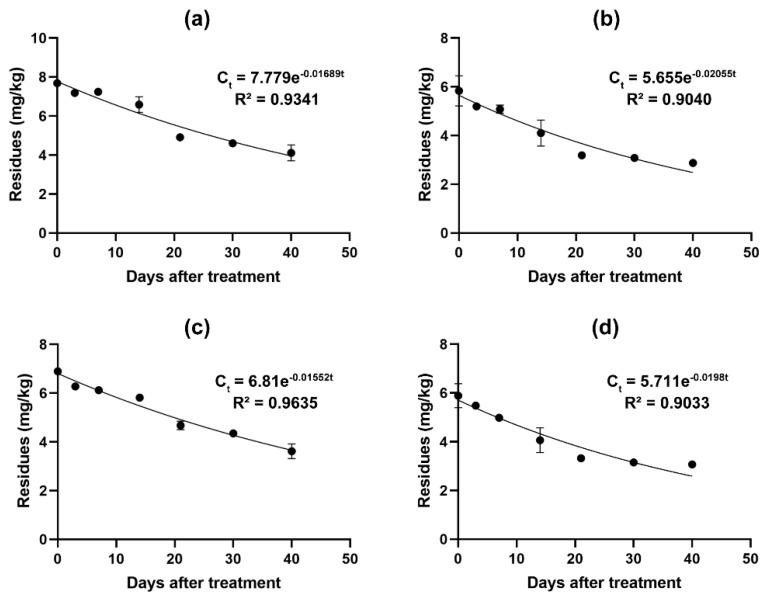
Dissipations of fludioxonil in cherry samples from (**a**) Anhui, (**b**) Beijing, (**c**) Henan, and (**d**) Shandong.

Figure 3 shows the curve of dissipation of fludioxonil with time in cherries from (a) Anhui, (b) Beijing, (c) Henan, and (d) Shandong. The curve indicates that the dissipation of fludioxonil in cherries followed first order kinetics. The half-lives of fludioxonil in cherries were 41 days, 33.7 days, 44.7 days and 35 days, and our results were different from the finding reported by Zhang that the half-lives of fludioxonil were 6.2–7.2 days in grapes and 6–12.1 days in soil [23]. Compared with our indoor low-temperature and light-proof environment, after applying pesticides in grape fields, the effects of temperature, light, and precipitation may accelerate the degradation process. Volatilisation, wash-off, plant growth, photolysis, pesticide physicochemical properties, chemical decomposition, and metabolism due to oxidation and hydroxylation, are all factors that play important roles in the limitation of pesticide residues in plants [46,47]. In addition, the activity of microorganisms in the soil and the adsorption of organic matter can also help degrade residual pesticides [48]. This can also explain why the residual level and half-life in our experimental results are higher than the previous research results of Zhang et al.

This study found that after using fludioxonil on chrysanthemums under outdoor conditions, the half-life of the fungicide is only 5.5 days [15], which is similar to the results of field experiments in grapes. It also shows that the dissipation rate of fludioxonil under outdoor conditions is much faster than indoors. In this study, after the application of fludioxonil, the cherries were stored in a low temperature environment so that the fungicide could exist stably for a long time and achieve better antibacterial and fresh-keeping effects. Studies have found that fludioxonil played an important role in the preservation of mango and avocado fruits [49,50]. Therefore, we speculate that fludioxonil may be more suitable as a low-temperature storage fungicide after fruit harvest than in field cultivation.

### 2.6. Terminal Residues and Dietary Risk Assessment

The quality control (QC) for real sample testing is carried out in Table 4. The average recoveries of fludioxonil in cherries were 80–101%, and the relative standard deviations (RSDs) were less than 4.2%. These data show that the detection method applied is stable and accurate.

Under the recommended dosage, the terminal residues of fludioxonil in cherries at 40 days were 3.84, 2.64, 3.28, and 2.58 mg/kg (Table 5), respectively, which are below the MRL (5.00 mg/kg) stipulated by China [40] and the European Commission [51]. Previous research found that residue levels in nectarines, apricots, and peaches after treatment with 100 mg/L fludioxonil at 20 °C averaged approximately 0.6–2 mg/kg [17]. Studies have shown that the degradation rate and residues of pesticides are usually closely related to the fruit size, properties of the epidermis, the water and sugar content, and others [52,53]. At the same time, these characteristics also determine the difficulty of fruit storage to a certain extent [54].

This study found that the terminal residues of fludioxonil on the three different types of cherries were different. Among them, the terminal residue and half-life of fludioxonil in the *Hongdeng* cherries were significantly higher than the others, indicating that the retention time of the fungicide in the *Hongdeng* cherries was longer and fludioxonil could play a good preservation effect. The fludioxonil residue level was closely related to fungicide concentration and treatment temperatures and was dependent on fruit species. Future research will focus on the relationship between different types of cherries and fludioxonil, as well as the most suitable treatment concentration and time. However, the residues of fludioxonil showed great persistence over through storage and shelf life [16,17]. Therefore, the recommendation is adequate storage time and necessary processes like washing to reduce its pesticide residue.

To evaluate the safety of consumers regarding pesticide residues, the exposure needs to be assessed and compared with health safety limits or toxicological endpoint values such as the acceptable daily intake and the acute reference dose. The dietary risk probability of fludioxonil was assessed via RQs, which were calculated by comparing the value of NEDI of fludioxonil with ADI. Assessment of the dietary risk to pesticide residues combines data on residues in foodstuffs with the data of food consumption. The average body weight (bw) of the general population was 63 kg in China and the ADI of fludioxonil formulated by EFSA was 0.37 mg/kg bw [55]. According to the above calculation method, the corresponding NEDI value calculated from the reference residue limit of the maximum dietary risk is 0.15473 mg (Table 6), which is far less than the ADI value established by the European Union. The STMR of fludioxonil concluded from the field trials was 3.35 mg/kg and was the reference residue limit of the evaluated cherries. As shown in Table 6, the RQ of fludioxonil was 0.61%. Hence, the above results indicate that the application of fludioxonil in cherries with the recommended dosage will not bring potential dietary risk for Chinese consumers.

## 3. Materials and Methods

### 3.1. Chemicals and Reagents

The standard of fludioxonil (C_12_H_6_F_2_N_2_O_2_, CAS: 131341-86-1, purity 99.1%) was purchased from Beijing Qincheng Yixin Technology Development Co., Ltd. (Beijing, China). Acetic acid, sodium chloride (NaCl), and anhydrous magnesium sulfate (MgSO_4_) were analytical grade, and the acetic acid was purchased from the Chemical Plant of Beijing and the others from Shanghai Aladdin Bio-Chem Technology Co., LTD. The acetonitrile in this study was both analytical grade and HPLC-grade provided by the Tianjin Jinke Fine Chemical Research Institute (Tianjin, China) and Beijing Mairuida Technology Co., Ltd. (Beijing, China). The HPLC-grade ammonium acetate was obtained from Beijing Dikma Technology Co., Ltd. (Beijing, China). Graphitized carbon black (GCB) and C_18_ were obtained from Tianjin Agela Tcehnologies Co., Ltd. (Tianjin, China). N-(n-Propyl) ethylenediamine (PSA, 40–60 μm), multiwalled carbon nanotubes (MWCNTs), and the syringe filters (nylon, 0.22 μm) were purchased from Bonna-Agela Technologies Venusil Technology Co., Ltd. (Tianjin, China).

The standard stock solution of fludioxonil (500 mg/L) and the secondary stock solution (100 mg/L) were prepared in HPLC-grade acetonitrile. The standard solution of fludioxonil (10 mg/L) was prepared in a volume of 50 mL by transferring 5 mL from the secondary stock solution. The solution was then serially diluted with HPLC-grade acetonitrile to obtain 0.1, 0.5, 1.0, and 5.0 mg/L series of standard solutions, all of which were stored at 4 °C until use.

### 3.2. Sample Preparation

**Figure 4:**
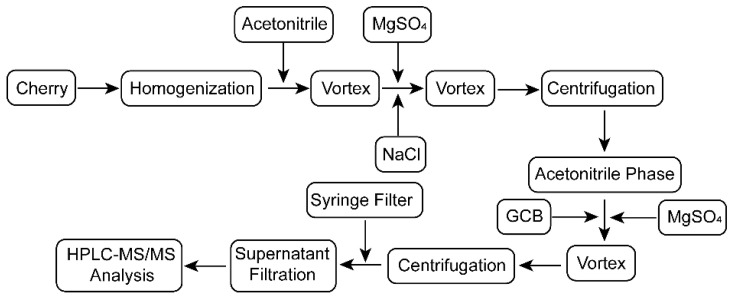
Flow chart of the extraction and purification process of the cherry samples.

In the QuEChERS method, the pits, stems and damaged fruits in the cherry samples were removed, and the pulp was chopped and homogenized. Cherry samples, 10 g each, were placed in 50 mL polypropylene centrifuge tubes and homogenized after adding 10 mL of acetonitrile. The samples were shaken vigorously by vortexer for 1 min until uniform, after which 4 g MgSO_4_ and 1 g NaCl was added, and the sample was again shaken vigorously by the vortexer for 1 min. The samples were centrifuged at a speed of 3000 rpm for 3 min. The 1.5 mL supernatant aliquot was transferred into a 3 mL centrifuge tube without disturbing the sediment, and the clean-up reagents of anhydrous MgSO_4_ (100 mg) and GCB (10 mg) were added. The mixture was vortexed for one min and then centrifuged at 10,000 rpm for 3 min. The clear supernatant extraction was filtered into an autosampler vial through a 0.22 μm syringe filter, and it was then analyzed via HPLC-MS/MS. The process of extraction and purification are shown in Figure 4.

### 3.3. Field Experiment Details

To study the dissipation and distribution of fludioxonil as a preservative in cherries, we designed field trials in four main cherry production areas in China: Beijing from 13 June 2018 to 23 July 2018 (40.45° N, 115.98° E, temperate monsoon climate, *Zaodaguo*); Suzhou city in Anhui province from 22 June 2018 to 1 August 2018 (33.65° N, 116.96° E, temperate monsoon climate, *Hongdeng*); Laiyang city in Shandong province from 16 June 2018 to 26 July 2018 (36.98° N, 120.72° E, temperate continental monsoon climate, *Meizao*); and Yongcheng city in Henan province from 27 June 2018 to 6 August 2018 (35.30° N, 113.93° E, temperate continental climate, *Hongdeng*). The field trials were designed in accordance with NY/T 788-2018 (*Guidelines on Pesticide Residue Trials*) issued by the Ministry of Agriculture, P. R. China [39]. The harvested cherry samples were used to measure the degradation dynamics and residues of fludioxonil.

On the day of cherry harvest, the cherries were soaked in the recommended dosage of 400 mg a.i./kg fludioxonil water solution for 1 min, then removed and dried at 0–4 °C. The processed cherry samples were put into fresh-keeping bags and stored at the storage temperature (−0.5 ± 0.5 °C). The control samples were treated with clean water. The cherry samples were collected at 0 (2 h after soaking), 3, 7, 14, 21, 30, and 40 days after soaking. The samples were then immediately analyzed by HPLC-MS/MS. All samples were stored −20 °C prior to further analysis.

### 3.4. HPLC Analysis

A high-performance liquid chromatography system (Agilent 1260, Santa Clara, CA, USA) tandem mass spectrometer (Agilent 6460, Santa Clara, CA, USA) equipped with an Agilent EC-C18 column (50 mm × 3 mm I.D., 2.7 μm) was employed to separate and quantify the fludioxonil simultaneously The oven temperature was set at 30 °C. The mobile phase of the acetonitrile (A) and 0.2% the acetic acid-5 mM/L ammonium acetate (B) was established with the volume ratio of 90:10 (*v/v*), and the flow rate was 0.3 mL/min. The sample injection volume was 10 μL. The parameters of MS detection were as follows: a gas temperature of 350 °C; a gas flow rate of 11 L/min; nebulizer gas pressure of 45 psi; and a column temperature of 30 °C. The capillary voltages were controlled at 4000 V. The analytes were determined in multiple reaction monitoring (MRM) mode. Under the operating conditions above, the simultaneous quantification of fludioxonil was performed based on the acquisition parameters as listed in Table 1.

### 3.5. Recovery Experiments

The standard solution was injected into the blank cherry sample with added concentrations of 0.01, 1, and 5 mg/kg, respectively. Five parallel treatments were performed at each spiked-level, and the recovery and relative standard deviation (RSD) was measured and calculated using the above analysis method.

### 3.6. Matrix Effect

The presence of matrix co-extracts after sample preparation frequently affects the signal response in a detector, which is known as “matrix effects”. The matrix effect may interfere with the accuracy of the analytical method to measure the quantification of the analyte, which causes errors in the quantitative or qualitative data, even leading to a false-negative or false-positive result [56]. Since complete elimination of the matrix effect is difficult in multi-residue analysis, the matrix-matched standard calibrations were used to calibrate possible interferences on the quantification of analytes as a compensatory strategy of matrix effects to obtain more realistic and valid results [34].

### 3.7. Statistical Analysis

The dissipation rate and half-life (t_1/2_) of fludioxonil in the cherries was evaluated by subjecting the data to a first-order kinetics equation:

C_t_ = C_0_e^−kt^, (1)

where C_t_ represents the concentration (mg/kg) of fludioxonil residue at time (t), C_0_ represents the initial concentration (mg/kg) of fludioxonil residue after application, and k is the dissipation coefficient in day 1. The persistence of fludioxonil is generally expressed in terms the of t_1/2_ or DT_50_, i.e., time for the disappearance of pesticide to 50% of its initial concentration and was calculated from the k value as following:

t_1/2_ = ln2/k, (2)

### 3.8. Dietary Risk Assessment

The national estimated daily intake (NEDI) for long-term intake risk and the risk quotient (RQ) was calculated by the following formulas [57]:

(3) $$\mathrm{NEDI}=\sum \frac{{\mathrm{STMR}}_{i}}{\mathrm{STMR}-P_{i}}\times F_{i},$$

(4) $$\mathrm{RQ}=\frac{\mathrm{NEDI}}{\mathrm{ADI}}\times \mathrm{bw},$$

where STMRi (mg/kg) represented the supervised trials median residue of fludioxonil in cherries in China. STMR-Pi was the supervised trials median residue corrected with the processing factor. Fi referred to the daily intake of a certain agricultural products or food in China (kg), ADI represented the acceptable daily intake, bw was the average body weight of a Chinese adult (63 kg) [58]. RQ was determined by comparing NEDI and ADI values and was usually determined under GAP. The higher the RQ value, the higher the pesticide residue; RQ > 100% means that the health risks of the evaluated food to consumers are unacceptably high [59].

## 4. Conclusions

In this paper, a confirmed reliable QuEChERS and a validated HPLC-MS method were constructed to detect the residues of fludioxonil in cherries. Samples were collected from four different locations and extracted with acetonitrile aqueous solution and purified with dispersive solid phase extraction. The half-lives of fludioxonil in the cherries were 33.7–44.7 days, and terminal residues were both below 5.00 mg/kg, which conformed to the temporary MRL of fludioxonil in cherries set by the European Commission. We speculate that fludioxonil may be more suitable as a low-temperature storage fungicide after fruit harvest than when applied during field cultivation. Additionally, the RQ values revealed that the associated risk of fludioxonil in cherries for Chinese consumers is extremely low. This current study validated that the application of the commercial fludioxonil (50% SE) in cherries as an indoor preservative is relatively safe at the recommended dosage according to the GAP conditions.

## Figures and Tables

**Table 1:** Acquisition parameters of fludioxonil in multiple reaction monitoring (MRM) mode.

Compounds	Retention Time (min)	Production (*m*/*z*)		Fragmentor (V)	Collision Energy (eV)	Polarity
		**Qualitative Ion**	**Quantitative Ion**			
Fludioxonil	1.00	247/180.1	247/126	160	180.1/3 126/10	Negative

**Table 2:** Recoveries (*n* = 5) of fludioxonil in cherry samples.

Matrices	Spiked Level (mg/kg)	Average Recoveries (%) N = 5	RSD (%)
Cherry	0.01	81	11.9
	0.5	91	3.7
	5	94	2.5

“*n* = 5” represents five repetitions for each level.

**Table 3:** Comparison of the presented method with other methods.

Method	Sample	Linear Range	LOD	LOQ	Extraction Recovery (%)	RSD (%)	Ref.
LLE-GC-MSD	white grape juice	0.01–1 mg/L	0.007 mg/L	0.013 mg/L	97–109	<15	[19]
LLE-HPLC-DAD	wine	0.17–20 mg/L	0.17 mg/L	0.17 mg/L	98.8	5.0	[18]
QuEChERS-LC-MS/MS	strawberry	0.002–0.5 mg/kg		0.002 mg/kg	95–116	5–12	[42]
Dilution-cELISA	apple juice	0.005–5 mg/L	0.00006 mg/L	0.005 mg/L	105–118	4–19	[20]
QuEChERS-GC-NPD	grape	0.1–10 mg/kg	0.03 mg/kg	0.1 mg/kg	94.35–100.89	0.67–6.67	[23]
QuEChERS-GC-MS	grape	0.02–2 mg/kg	0.006 mg/kg	0.02 mg/kg	83.6–97.67	1.84–10.31	[13]
QuEChERS-UPLC-MS/MS	chrysanthemum	0.005–0.2 mg/kg		0.005 mg/kg	91.36–107.85	0.05–10.35	[15]
QuEChERS-HPLC- MS/MS	cherry	0.005–5 mg/kg	0.005 mg/kg	0.01 mg/kg	81–94	2.5–11.9	This method

**Table 4:** Quality control (QC) of real sample detection.

Matrix	Date of Detected	Spiked Level (mg/kg)	Compounds	Average Recovery (%)	RSD (%)
Cherry	7 May 2019	5.0	Fludioxonil	80	3.5
Cherry	9 May 2019	5.0	Fludioxonil	101	4.2

**Table 5:** Terminal residues of fludioxonil in cherry samples.

Location	Species	Dose (mg a.i./kg)	Days after Spraying	Terminal Residue (mg/kg)
Suzhou city of Anhui province	*Hongdeng*	400	30	4.17
			40	3.84
Beijing	*Zaodaguo*		30	2.55
			40	2.64
Yongcheng city of Henan province	*Hongdeng*		30	4.11
			40	3.28
Laiyang city of Shandong province	*Meizao*		30	3.49
			40	2.58

**Table 6:** The chronic dietary intake risk assessment of fludioxonil in accordance with Chinese dietary patterns.

Food Classification	Fi (kg)	Reference Residue Limits or STMR	Sources	NEDI (mg)	ADI (mg)	Risk Quotient (%)
Rice and its products	0.2399				ADI × 63	
Flour and its products	0.1385					
Other grains	0.0233					
Tubers	0.0495					
Dried beans and their products	0.016					
Dark vegetables	0.0915					
Light vegetable	0.1837					
Pickles	0.0103					
Fruits	0.0457	3.35	STMR	0.153095		
Nuts	0.0039					
Livestock and poultry	0.0795					
Milk and its products	0.0263					
Egg and its products	0.0236					
Fish and shrimp	0.0301					
Vegetable oil	0.0327	0.05	China	0.001635		
Animal oil	0.0087					
Sugar, starch	0.0044					
Salt	0.012					
Soy sauce	0.009					
Total	1.0286			0.15473	25.2	0.61

STMRi (mg/kg) represented supervised trials median residue of fludioxonil in cherries in China, Fi referred to the daily intake of a certain agricultural products or food in China (kg), bw was the mean of the average body weight of Chinese adults (63 kg).

## Data Availability

Data are contained within the article.

## Sample Availability

Samples of the compound fludioxonil are available from the authors upon request.
